# Differences in MicroRNA Expression in Firefighters Responding to a Train Derailment and Fire in East Palestine, Ohio

**DOI:** 10.3390/epigenomes10010008

**Published:** 2026-02-03

**Authors:** Jaclyn M. Goodrich, Yaodong Xin, Shawn C. Beitel, John Gulotta, Lu Wang, Bhavya Thotakura, Judith M. Graber, Derek Urwin, Alexander C. Mayer, Sara Jahnke, Derrick L. Edwards, Casey Grant, Sreenivasan Ranganathan, Jefferey L. Burgess

**Affiliations:** 1 Department of Environmental Health Sciences, University of Michigan School of Public Health, Ann Arbor, MI 48109, USA; 2 Department of Biostatistics, University of Michigan School of Public Health, Ann Arbor, MI 48109, USA; yaodongx@umich.edu (Y.X.); luwang@umich.edu (L.W.); 3 Department of Community, Environment and Policy, Mel and Enid Zuckerman College of Public Health, University of Arizona, Tucson, AZ 85724, USA; sbeitel@arizona.edu (S.C.B.); jburgess@arizona.edu (J.L.B.); 4 Tucson Fire Department, Tucson, AZ 85701, USA; john.gulotta@tucsonaz.gov; 5 College of Osteopathic Medicine, Michigan State University, East Lansing, MI 48824, USA; thotaku1@msu.edu; 6 Department of Biostatistics and Epidemiology, Rutgers, The State University of New Jersey, Piscataway, NJ 08854, USA; judith.graber@rutgers.edu; 7 Department of Chemistry and Biochemistry, University of California, Los Angeles, CA 90095, USA; djurwin@ucla.edu; 8 Los Angeles County Fire Department, Los Angeles, CA 90063, USA; 9 Division of Field Studies and Engineering, National Institute for Occupational Safety and Health, Centers for Disease Control and Prevention, Cincinnati, OH 45226, USA; nru1@cdc.gov; 10 NDRI-USA, Leawood, KS 66224, USA; jahnke@ndri-usa.org; 11 Department of Counseling and Psychology, Tennessee Tech University, Cookeville, TN 38505, USA; dedwards@tntech.edu; 12 Fire Protection Research Foundation, Quincy, MA 02169, USA

**Keywords:** high-risk incidents, fire service, occupational health, disaster response, epigenetics, noncoding RNA, biomarkers

## Abstract

**Background/Objectives**: High-risk, low-frequency incidents such as building collapses and large chemical fires can result in acute, high-dose exposures to toxic agents for first responders and the surrounding community. While these exposures may last for hours to days, their contribution to firefighters’ risks for cancer and other diseases is relatively unknown. In February 2023, a freight train transporting chemicals derailed and caught fire in East Palestine, Ohio, US. More than 350 firefighters, primarily volunteer, responded to the incident. In this cross-sectional study, we evaluated epigenetic markers of toxicity in responding firefighters. We hypothesized that exposures from responding to the train derailment would alter the expression of microRNAs (miRNAs) linked to carcinogenesis. **Methods**: We enrolled 62 responding firefighters and a comparison group of 26 firefighters from the same region who did not respond to the incident. We measured the relative expression of 800 miRNAs in blood samples using the nCounter Human v3 miRNA expression panel. We compared the expression of miRNA between exposure groups in negative binomial regression models, adjusting for potential confounders. **Results**: At a false discover rate cut-off of 5% (q-value < 0.05), 16 miRNAs had significantly higher expression and one significantly lower among firefighters that responded to the incident. Top disease-related pathways in which these miRNAs were enriched included those relevant to neurodegenerative diseases, vascular disease, and multiple cancer sites. **Conclusions**: Overall, results suggest responding to one large incident can have non-transient impacts on miRNA expression. Whether this translates into longer-term health risks or adaptive responses to exposures is unclear.

## 1. Introduction

Firefighters are at increased risk for several cancers compared to the general population. Based on the evidence from epidemiological and mechanistic studies, the International Agency for Research on Cancer declared occupation as a firefighter to be a group 1 carcinogen [1]. This may be due to the combination of carcinogenic exposures firefighters encounter over years of work, including exposure to polycyclic aromatic hydrocarbons, per- and polyfluoroalkyl substances, diesel, volatile organic compounds, and more [2,3,4,5]. Occupational stressors like sleep disruption, excess alcohol consumption, and heat stress may also contribute to adverse health outcomes [6,7]. It is unclear the extent to which specific chemicals or other occupational hazards contribute to firefighters’ health risks. While it is expected that exposure burden accumulating over years contributes to disease, it is unknown whether single large dose exposure incidents have a substantial contribution to firefighter health risks.

Incidents such as large chemical fires, building collapses, hurricanes, and multi-day wildland/urban interface (WUI) fires can result in acute, high-dose exposures to toxic chemical or biological agents. Volunteer and career firefighters alike may need to respond to these high-risk, low-frequency incidents during the course of their careers. While these incidents are rare, response can involve hundreds to thousands of firefighters and encompass an acute exposure burden that lasts for hours, days, or even weeks. Given the duration of the response, it is not always feasible to don the appropriate personal protective equipment (PPE), including respiratory protection, for the entire response. To date, the primary methods for estimating firefighter cancer incidence are retrospective cohort studies, which lack detailed exposure information and, therefore, cannot be used to determine the contribution of these incidents to overall risk for cancer or other health issues that firefighters face. There has been ample research on responders to the World Trade Center attack in the US due to a long-term health monitoring program that was established following the incident. Research to date has shown responders to this incident are at increased risk for prostate and thyroid cancers [8], long-term lung function decline and bronchial hyper-reactivity [9,10], and dementia [11,12]. Health monitoring after other high-risk incidents is important since the exposure burden (mixture of chemicals, dose, and duration) varies by incident. Even so, exposure and health measures among firefighters have been monitored after a few high-risk incidents.

Cancer is a multistage process with a latency period between exposure and the onset of disease from as short as several years to over 30 years [13]. Molecular biomarkers, such as epigenetic alterations, reflect subtle biological changes that occur following exposures that can contribute to subsequent disease development. Epigenetic alteration serves as a subtle indicator, or effect biomarker, of toxicity that is present long before development of overt disease [14]. Epigenetic modifications include non-coding RNA expression such as miRNAs, DNA methylation, and histone modifications. The epigenome controls normal cellular function through regulating gene expression. Aberrant epigenetic modifications contribute to nearly all known disease processes, including cancers [15,16,17,18]. In terms of carcinogenesis, there is an epigenetic contribution to nearly every hallmark of cancer, and epigenetic modification is considered to be one of the key characteristics by which carcinogenic chemicals exert their effects [14,19,20]. While the epigenome differs by cell and tissue type, blood is commonly used as a surrogate tissue in epidemiological studies. Measurement of miRNAs in blood can discriminate between patients with some cancers and controls [21]. MiRNAs are small non-coding RNAs that regulate gene expression, specifically suppressing translation of messenger RNA. Altered miRNA expression affects cell cycle and survival programs and has been reported in a variety of cancers [22].

We previously identified miRNA differences between new firefighters and incumbents. Additionally, we observed changes in miRNA among new firefighters from the beginning of their careers to two years into their service. These findings suggest that cumulative workplace exposures influence miRNA expression [23,24]. We also identified 50 miRNAs that changed over ten months among firefighters who responded to at least one WUI fire between the baseline and follow-up visits [25]. This study suggested that exposures from WUI fires may influence miRNA expression. Whether these alterations are adaptive or harmful and whether they are transient or persistent is currently unknown.

Chemical-based fires, while rare, may pose a significant threat to community and firefighter health. In 2023, a train transporting chemicals, including vinyl chloride, butyl acrylate, and benzene, derailed and caught fire in East Palestine, Ohio (US). This rural community is served primarily by volunteer fire departments. Local volunteer firefighters responded to the incident first, without awareness or preparation for the toxicants they were being exposed to on the first night of the response. To prevent an explosion, there was a controlled burn of the vinyl chloride-containing cars on February 6, with all fire extinguished by February 8. Overall, over 350 firefighters responded to the incident from more than 50 departments in the surrounding region. Vinyl chloride and benzene are both group 1 carcinogens [26,27]. Vinyl chloride has been linked to genotoxicity and miRNA alterations in a previous occupational exposure study [28].

Given potential high-dose exposures to vinyl chloride and other carcinogens over hours to days, we hypothesized that the expression of cancer-related miRNAs would be altered in firefighters who responded to the incident. We utilized study protocols from the national Fire Fighter Cancer Cohort Study (FFCCS) [29] to enroll firefighters 2–4 months after the incident, collect blood, and measure the expression of 800 miRNAs in firefighters who responded to the fire and a group of firefighters from the same region who did not.

## 2. Results

### 2.1. Descriptive Statistics

Out of the estimated >350 firefighters that responded to the East Palestine derailment, 62 participated in our study. We recruited a comparison group of 26 firefighters from the same region who did not respond to the incident. The two groups had similar age and BMI distributions. The majority of both groups were volunteer firefighters, white non-Hispanic males, and never-smokers (Table 1). The notable exception was firefighter years, as the incident responder group reported working as a firefighter for 19.8 years on average (sd = 13.1) compared to 13.3 years (sd = 12.6) in the comparison group.

### 2.2. miRNA Expression: Comparing Exposure Groups

**Figure 1:**
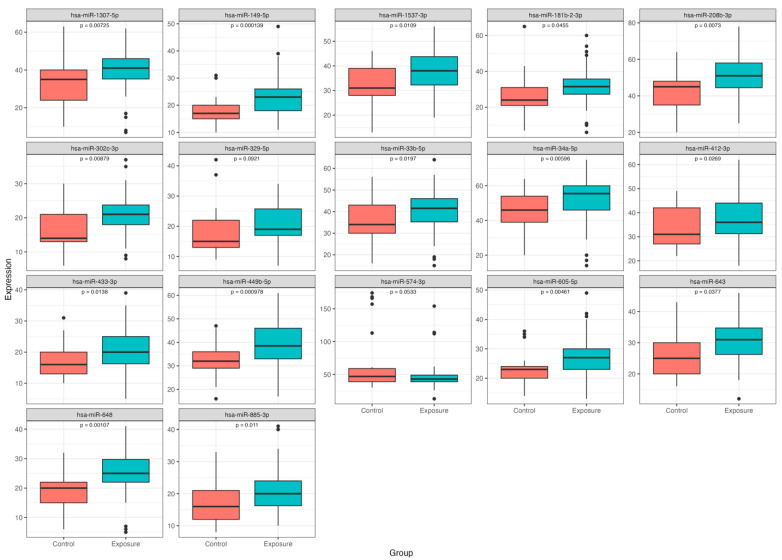
Differentially expressed microRNA. Expression of ~800 miRNA in blood samples was compared between firefighters responding to the East Palestine train incident and a control group of firefighters who did not. After adjusting for covariates, these 17 miRNAs were significantly different between groups (q < 0.05). Boxplots describe the relative abundance of the significant miRNA between the exposed group (‘exposure’; blue) and the comparison group (‘control’; orange). Units of expression are read counts after normalization by the Remove Unwanted Variation using control genes (RUVg) method. Raw *p*-values for a *t*-test comparing expression levels between the two groups are shown on the plot. All miRNAs were significantly differentially expressed in models adjusted for covariates at a q-value < 0.05 (Supplemental Table S1).

A total of 17 miRNAs were differentially expressed by incident response status (q < 0.05). Sixteen had increased expression in firefighters who responded to the incident relative to the comparison firefighter group (Supplemental Table S1; Figure 1). One miRNA, hsa-miR-574-3p, had lower expression in the exposed group compared to the comparison group in adjusted models.

It is unknown whether the expression of similar miRNAs is altered in response to different fire-related exposures. We cross-referenced miRNAs that changed over a ten-month period among firefighters responding to at least one WUI fire in our previous study (Supplemental Table S2) [25]. There were 50 significant miRNAs in the previous study, of which 49 were in our dataset, and 5 varied in the exposed group within our study (*p* < 0.1 and q < 0.2). Only one was in the same direction. The hsa-miR-660-3p expression was increased in the exposed group in this study, and also increased at follow-up months after a WUI fire response in the previous study.

The East Palestine incident consisted of a unique set of exposures, including vinyl chloride, that are not common in other fires. One previous study of industrial workers exposed to vinyl chloride for at least one year evaluated the association of this exposure with miRNA expression. Of the 15 associated with exposure in the previous study, 12 were assayed in samples from the firefighters of our study (Supplemental Table S3). A total of 2 of the 12 had differences in expression between the exposure groups of our study (q < 0.2): miR-144-3p and miR-151b.

### 2.3. Pathway Enrichment

We assessed whether the 17 miRNAs that significantly differed between exposure groups were overrepresented in biological or disease-relevant pathways using software that considers the downstream target genes of each miRNA (Table 2). Thirty-three disease pathways from the MNDR database were over-represented (q < 0.05). Significant pathways included neurological (brain disease, neurodegenerative disease, Alzheimer’s disease), and 22 cancer-related conditions, including breast cancer, lung cancer, hepatocellular carcinoma, prostate cancer, melanoma, and more.

## 3. Discussion

We recruited firefighters who responded to the East Palestine train derailment in 2023, a high-risk, low-frequency incident that resulted in multi-day exposure to vinyl chloride, benzene, and other toxic chemicals. When comparing miRNA expression in blood samples from 62 firefighters exposed to the incident to blood from 26 comparison firefighters from the same region, we identified 16 miRNAs with higher expression and 1 with lower expression in the exposed group. Since samples were collected two to four months after the incident response, this may reflect a prolonged response to the exposures. MiRNAs were enriched in neurodegenerative and cancer-related disease pathways.

MiRNAs play essential regulatory roles in physiological processes. These non-coding molecules have been linked to processes such as cell proliferation, differentiation, and apoptosis. Some act as tumor suppressors and others as promoters, depending on timing and the cancer/tissue type. The miRNA with lower expression in the incident responder group—miR-574-3p—has been linked to various cancers in previous studies, often with a tumor suppressor role. For example, miR-574-3p expression is reduced in early-stage gastric cancer tissue [30]. In vitro, transfecting gastric cancer cells with this microRNA inhibits the proliferation and migration of the cells [30]. MiR-574-3p may also be a tumor suppressor in ovarian cancer. In an ovarian cancer cell line, overexpression of miR-574-3p reduced proliferation, invasion, and migration of the cells, and this occurred in part through its inhibition of matrix metalloproteinase 3 [31]. Decreased levels of miR-574-3p were measured in esophageal tumors, and miR-574-3p expression inhibited cancer cell proliferation in part through the mitogen-activated protein kinase 1 pathway [32]. Conversely, miR-574-3p levels were higher in serum microvesicles collected from patients with glioblastoma multiforme compared to controls [33]. This miRNA had higher expression in firefighters who responded to a WUI fire [25], suggesting this miRNA may be an exposure-sensitive biomarker that responds differently depending on the exposure and timing.

Higher expression of hsa-miR-149-5p was observed in the exposed group in this study. The hsa-miR-149-5p has been implicated in a wide range of disease processes, including various cancers and neurodegenerative diseases, and its expression varies across disease contexts. This miRNA is generally downregulated in prostate carcinoma, where it acts as a tumor suppressor by restraining the regulator of G protein signaling 17, which in turn impairs tumor angiogenesis, growth, and metastasis [34]. Similarly, hsa-miR-149-5p inhibits the progression of other tumor types, including renal cancer, melanoma, thyroid carcinoma, and colorectal carcinoma. However, hsa-miR-149-5p can also have pathogenic effects. For example, its upregulation in myeloid leukemia has been shown to promote disease progression by decreasing cell apoptosis via effects on Fas Ligand [35]. In addition, increased expression of hsa-miR-149-5p in bronchial premalignant lesions is associated with lesion progression, particularly in epithelial regions and areas of dysplasia. Its upregulation impairs antigen presentation and processing in airway basal cells, resulting in immune evasion [36]. Overall, hsa-miR-149-5p exhibits complex roles that can be either tumor-suppressive or oncogenic, depending on the cellular context.

Several disease pathways contained targets of nearly all (>70%) of the 17 significant microRNAs that were significantly overrepresented according to the MNDR database. These pathways involved neurological disorders (brain disease, vascular diseases, neurodegenerative diseases, and Alzheimer’s disease) and cancers (leukemia, carcinoma, lung cancer, breast cancer, and hepatocellular carcinoma). While disease causality cannot be inferred from altered expression of miRNA in a given pathway, these results build on established links between firefighting exposures and cancers [37] and a growing body of evidence that firefighting exposures are linked to neurodegenerative diseases. For example, cognitive impairment and dementia risk were increased among World Trade Center first responders [11,12]. In a study of Korean non-smoking male firefighters, urinary polycyclic aromatic hydrocarbon (a common exposure for firefighters) concentrations were associated with brain volume reduction in the frontal, parietal, temporal, and cingulate lobes. These brain changes are suggestive of neurodegeneration, mild cognitive impairment, and early-stage dementia in Alzheimer’s disease [38].

Vinyl chloride was an exposure at this high-risk incident that firefighters rarely encounter. However, the health impacts of vinyl chloride exposure have been demonstrated in previous studies of chronically exposed workers. Among vinyl chloride monomer-exposed workers, analysis of miRNA expression revealed 15 differentially expressed miRNAs in a discovery set of 12 exposed workers. Four of these miRNAs were validated in 94 exposed workers when compared to 53 office workers. Among the 15 differentially expressed miRNAs, miR-144-3p and miR-151b also differed between exposure groups in the current study (*p* < 0.05); however, the direction of association with exposure was in the opposite direction [28]. In a larger group of vinyl chloride-exposed workers, there was a significant dose–response relationship between exposure and micronuclei in blood lymphocytes, an indicator of chromosomal damage [39]. Exposed individuals also exhibited differential DNA methylation at >9000 regions, including in genes involved in cancer and neurological function [40]. Another study of polyvinyl chloride workers in Italy reported a 50% increased risk of lung cancer mortality among workers compared to the regional reference, with a doubled risk of lung cancer linked to both malignant and non-malignant lung diseases even after adjusting for smoking [41]. Finally, Schaffer et al. described three cases of rare hepatic angiosarcoma in workers at a US-based vinyl chloride monomer polymerization plant, which led to stricter regulations that prevented further cases after 1975 [42]. Together, these findings highlight that vinyl chloride exposure contributes to carcinogenic risks, potentially through mutagenic and epigenetic pathways, especially in the liver and lungs.

Benzene is another known carcinogen that firefighters may have been exposed to at the East Palestine train derailment. In a study of 247 benzene series (BTEX)-exposed workers compared to 256 non-exposed controls, serum expression of miR-181a-5p, miR-221-3p, miR-223-3p, and miR-342-3p was lower, and miR-638 was higher among the BTEX-exposed group [43]. With all five miRNAs, there was also a corresponding dose–response relationship within the BTEX-exposed worker group. These miRNAs were selected for analysis due to their abnormal expression in leukemia and/or correlation with benzene exposure in experimental models. Within our study, these miRNAs were not among the top hits (q < 0.05). However, three were differentially expressed in the expected direction at q < 0.2, suggesting that benzene may have played a role. MiR-221-3p and miR-223-3p were lower among responders to the East Palestine incident, and miR-638 expression was higher compared to the firefighters who did not respond.

While the mixture of exposures at this incident was unique, it is not known whether the molecular response to these exposures would be similar to those following other exposures. One miRNA was more highly expressed among responders to the train derailment and in firefighters responding to a WUI fire [25]—hsa-miR-660-3p. This miRNA may play a role in liver fibrosis, which is a risk factor for hepatocellular carcinoma and other liver cancers. In vitro, miR-660-3p was involved with the progression of liver fibrosis by promoting hepatic stellate cell (HSC) activation via the inactivation of telomerase-associate protein 1 involved in the maintenance of telomeres [44]. We also compared our results to past studies identifying miRNAs associated with cumulative structural firefighter exposures [23,24]. Two miRNAs associated with cumulative exposures in past studies were associated in the same direction with exposure in our study. The hsa-miR-548ad-3p expression was higher in incident responders in this study and positively associated with employment duration among new firefighters followed-up over time [23]. The hsa-miR-5010-3p was higher in incident responders in this study and in incumbents compared to recruits in Jeong et al. [24]. Thus, miRNA responses seem to be primarily exposure-specific, with a small set of miRNAs that are increased for multiple exposures (hsa-miR-660-3p, hsa-miR-5010-3p, and hsa-miR-548ad-3p).

This is one of the few studies to evaluate the biological impacts of firefighters responding to a high-risk, low-frequency incident. Strengths include the non-responder control group, which is overall similar in demographic and firefighter-related characteristics when compared to the responder group. By using this group as a reference, we reduce the chances of confounding from other firefighting-related exposures, regional environmental exposures, socioeconomic status, and demographic factors. Limitations of this study include the small sample size and lack of personal exposure information from firefighters who responded to the incident. Given the difference in total years of experience between groups, there is a chance that other firefighting exposures are confounding the results. A further limitation is the time lag between exposure and sampling, as well as the current availability of measures at one time point (with no baseline pre-exposure), and the samples available. We measured miRNA in blood as a biomarker of the body’s response to the exposures. MiRNA expression can be tissue-specific, and this does not necessarily reflect miRNA expression in target organs of interest, such as the liver. Even so, for some cancers, blood epigenetic markers are representative of markers in the affected tissue [45,46]. Future research should continue evaluating high-risk, low-frequency incidents, incorporating both baseline and follow-up miRNA expression monitoring. This approach will help assess the long-term health risks to firefighters associated with these incidents.

## 4. Materials and Methods

### 4.1. Cohort Recruitment and Study Population

In this cross-sectional study, we utilized the study protocols of the FFCCS to enroll participants, collect information on demographics and occupational practices, and collect blood and urine samples [29]. Enrollment events were held in locations near East Palestine, Ohio, in April and June of 2023. Firefighters in the region (volunteer, career, or both) were invited to participate, whether or not they responded to the incident. Firefighters who enrolled but were not present for the train fire response comprise the comparison group. Firefighters who responded during any part of the train fire (hours to days) were considered the event-response group. Self-administered online questionnaires collected information on demographics, general fire service information (i.e., years worked as a career and/or volunteer firefighter, current department, job type, PPE use, and other fireground behaviors), lifestyle information (i.e., tobacco and alcohol use, physical activity), and health information with a focus on cancers and family history of cancers. Blood and urine samples were collected by the trained study team.

All study protocols and materials were approved by the University of Arizona Institutional Review Board (IRB), the University of Michigan IRB, and the Centers for Disease Control and Prevention (CDC)/National Institute for Occupational Safety and Health (NIOSH). The IRB deferred oversight of the study to them. Study participants provided written informed consent.

### 4.2. MiRNA Analysis

For miRNA, sample collection and processing have been reported previously [23,24]. At the time of enrollment, venous blood was drawn via venipuncture and collected into Tempus^TM^ Blood RNA tubes (Applied Biosystems, Foster City, CA, USA). Tubes were shaken vigorously according to the manufacturer’s guidelines, and then aliquoted into 5 mL cryogenic tubes and stored at −80 °C. RNA was isolated according to the manufacturer’s recommendations using MagMAX^TM^ for Stabilized Blood Tubes RNA Isolation Kit (Life Technologies, Carlsbad, CA, USA).

MiRNA expression was measured using the nCounter^®^ Human v3 miRNA expression panel (NanoString Technology Inc., Seattle, WA, USA), to profile approximately 800 curated and clinically relevant human miRNAs from miRBase v21, 5 housekeeping genes, and 20 assay controls (6 positive, 8 negative, and 6 ligation controls).

### 4.3. Statistical Analysis

All data pre-processing and statistical analyses were conducted in the R Project for Statistical Computing (version >= 4.0.3). Descriptive statistics were calculated and compared between exposure groups when appropriate using t-tests or chi-square tests.

We aimed to identify miRNA expression counts that differed between firefighters who responded to the train derailment and those who did not. We first pre-processed the miRNA data to remove variation from technical sources, utilizing the Remove Unwanted Variation using control genes (RUVg) method [47]. This method performs factor analysis of the read counts based on a subset of negative control genes, which are known a priori not to be differentially expressed between the samples under consideration. Spike-in genes were used as negative controls in this process to ensure accurate normalization and adjustment for unwanted variation in the data.

To identify miRNAs that were differentially expressed between the two groups, we performed a t-test for each miRNA after processing. We next fit a negative binomial regression model for each miRNA in order to appropriately handle the overdispersion of the miRNA expression data [48]. The main predictor in these models was the firefighters’ response to the incident (yes/no). To account for potential confounding, we adjusted for sex, age, BMI, career type (volunteer versus career or career and volunteer), and smoking status (ever smoker vs. non-smoker; ever smoker was considered someone who reported smoking at least 100 cigarettes in their lifetime). Age and years of firefighting experience were highly correlated (correlation coefficient 0.8), and thus, we did not include both in the model due to multicollinearity. To adjust for multiple comparisons, we calculated the FDR q-values based on the *p*-values of the response predictor in each regression model. A q-value ≤ 0.05 was considered statistically significant. We cross-referenced whether statistically significant miRNAs were also associated with firefighter exposure to a WUI fire in our previous study [25] or with vinyl chloride exposure in a study of occupational exposure [28].

We conducted pathway enrichment analysis using the subset of miRNAs with q-values ≤ 0.05. Over-representation analysis was performed using the miEAA (miRNA Enrichment Analysis and Annotation) tool version 2.1 [49]. The databases of miEAA we used included KEGG, miRWalk (pathways and diseases), Mammalian ncRNA Disease Repository (MNDR), and Gene Ontology from annotations derived from miRTarBase. Pathway enrichment analysis helped us to identify biological pathways that are significantly associated with the differentially expressed miRNAs and their downstream target genes. We conducted over-representation analysis in these pathway sets and considered pathways with q < 0.05 to be statistically significant. Using miEAA [49,50], we also cross-referenced disease annotations from human and experimental studies for statistically significant miRNA to infer function and disease relevance via HMDD v.0 (Human microRNA Disease Database, https://www.cuilab.cn/hmdd; accessed 1 April 2024 [51]).

## 5. Conclusions

We identified miRNAs that were associated with response to the East Palestine train derailment, a high-risk, low-frequency incident that resulted in exposures to vinyl chloride, benzene, and other toxicants. While it is known that the occupation of firefighting is linked to increased cancer risk [37], whether some exposures—especially those from acute high-exposure incidents—contribute substantially to this risk is not known. Ours is one of the few studies to follow-up on health biomarkers in firefighters who responded to a high-risk incident. Results suggest that a few miRNAs respond to multiple exposures, while others are exposure-specific. Whether these miRNA expression differences persist years beyond the incident is the focus of ongoing research and will help to inform whether these alterations are merely adaptive or could contribute to long-term disease development.

## Figures and Tables

**Table 1:** Characteristics of the Study Population.

		All Participants (*n* = 88)		Responded to the East Palestine Incident (*n* = 62)		Did Not Respond (*n* = 26)		
		***n***	***%***	***n***	***%***	***n***	***%***	p-Value *
Career Type	Career	11	12.5%	8	12.90%	3	11.5%	
	Volunteer	67	76.1%	46	74.20%	21	80.8%	
	Both	10	11.4%	8	12.90%	2	7.7%	0.92
Sex	Male	75	85.2%	55	88.70%	20	76.9%	
	Female	13	14.8%	7	11.30%	6	23.1%	0.19
Smoking Status	Current	11	12.5%	9	14.50%	2	7.7%	
	Past	11	12.5%	9	14.50%	2	7.7%	
	Never	66	75.0%	44	71.00%	22	84.6%	0.53
		***mean***	***SD***	***mean***	***SD***	***mean***	***SD***	
Age (years)		41.3	14	41.9	13.4	40	15.7	0.6
BMI (kg/m^2^)		31.4	6.9	32	7.2	29.8	5.7	0.14
Firefighting Years		17.9	13.2	19.8	13.1	13.3	12.6	0.03

* Test comparing the two exposure groups.

**Table 2:** Over-represented pathways among miRNAs that differed between exposure groups from the MNDR database.

Subcategory	*p*-Value	Q-Value	Expected Number of miRNA	Observed Number of miRNA in this Pathway
Brain disease	2.39 × 10^−5^	0.007	4.5	13
Vascular diseases	1.41 × 10^−5^	0.007	5.2	14
Leukemia	4.83 × 10^−5^	0.007	4.8	13
Neurodegenerative diseases	4.17 × 10^−5^	0.007	4.7	13
Carcinoma	1.52 × 10^−4^	0.009	4.4	12
Eye disease	1.31 × 10^−4^	0.009	3.6	11
Lung cancer	1.28 × 10^−4^	0.009	4.4	12
Melanoma	1.57 × 10^−4^	0.009	3.7	11
Prader–Willi syndrome	1.24 × 10^−4^	0.009	0.0	2
Progesterone receptor positive breast cancer	1.41 × 10^−4^	0.009	4.4	12
Breast cancer	1.80 × 10^−4^	0.009	6.3	14
Prostate cancer	1.96 × 10^−4^	0.009	3.8	11
Hepatocellular carcinoma	2.16 × 10^−4^	0.009	5.5	13
Breast carcinoma	2.84 × 10^−4^	0.011	1.5	7
Bone disease	3.87 × 10^−4^	0.014	3.3	10
Familiar ovarian carcinoma	4.36 × 10^−4^	0.014	3.4	10
Li–Fraumeni syndrome	4.11 × 10^−4^	0.014	0.0	2
Diabetes mellitus	6.27 × 10^−4^	0.019	3.5	10
Malignant glioma	9.26 × 10^−4^	0.027	3.7	10
Solid-pseudopapillary neoplasm of pancreas	0.001	0.032	1.8	7
Alzheimer’s disease	0.001	0.038	5.6	12
Cervical squamous cell carcinoma	0.002	0.038	1.4	6
Glioblastoma	0.002	0.038	4.7	11
Acute myelocytic leukemia	0.002	0.040	0.6	4
Estrogen-receptor-negative breast cancer	0.002	0.040	4.0	10
Lung adenocarcinoma	0.002	0.040	4.8	11
Lymphoma	0.002	0.040	3.3	9
Pituitary neoplasms	0.002	0.040	1.5	6
Familial ovarian cancer	0.002	0.044	3.4	9
Progesterone receptor negative breast cancer	0.002	0.045	4.1	10
Liver cirrhosis	0.003	0.049	0.6	4
Osteosarcoma	0.003	0.049	2.8	8
Skin disease	0.003	0.049	1.5	6

## Data Availability

Data are available upon reasonable request. Data requests will be reviewed by the study’s Oversight and Planning Board to address firefighter concerns prior to determination of sharing de-identified data. Contact the corresponding author with requests.

## Supplementary Materials

The following supporting information can be downloaded at: https://www.mdpi.com/article/10.3390/epigenomes10010008/s1, Supplemental Table S1: Association between East Palestine incident response and miRNA expression in adjusted negative binomial regression models; Supplemental Table S2: Association between East Palestine incident response and miRNA expression at miRNA associated with WUI fire response in a past study; Supplemental Table S3: Association between East Palestine incident response and miRNA expression at miRNA associated with vinyl chloride among workers in a past study.

## Abbreviations

The following abbreviations are used in this manuscript:

WUI	Wildland/urban interface
FFCCS	Firefighter Cancer Cohort Study
miRNA	microRNA
miEAA	microRNA Enrichment Analysis and Annotation
BMI	Body mass index
